# An algorithmic approach in cases of dual diagnosis - gaming disorder and major depressive disorder

**DOI:** 10.1192/j.eurpsy.2026.10801

**Published:** 2026-07-31

**Authors:** O. Vasiliu, A. M. Ciobanu

**Affiliations:** 1 Clinical Neurosciences Department, “Carol Davila” University of Medicine and Pharmacy; 2 Psychiatry Department, “Dr. Carol Davila” University Emergency Central Military Hospital; 3 ”Prof.dr. Al. Obregia” Psychiatry Clinical Hospital, Bucharest, Romania

## Abstract

**Introduction:**

The need to approach systematically the cases of bipolar disorder (BD) and gaming disorder (GD) from a diagnosis and therapeutic perspective is supported by the potential negative consequences of this comorbidity on the evolution and prognosis. Although there are authors who critically evaluate this dual diagnosis, given the potential for high rates of confounders from a clinical perspective, a structured approach to this phenomenon is necessary. This necessity is based on the fact that early detection and intervention in cases of dual diagnoses are expected to improve the chances of these patients to achieve the remission of symptoms, functional recovery, and a better quality of life.

**Objectives:**

The main aim of this review was to find in the literature data for the development of an algorithmic approach for screening, monitoring, and therapy in patients with comorbid BD and GD.

**Methods:**

A literature review was conducted in three electronic databases (PubMed, EMBASE, and Clarivate/Web of Science) using the keywords „bipolar disorder,” „behavioral addiction,” „gaming disorder,” and „online gaming disorder” for papers published in English between January 2000 and February 2025. Both primary and secondary reports are needed.

**Results:**

No structured interventions exist for the screening and/or treatment of cases when both BD and GD are diagnosed, which leaves it to the clinicians to justify their therapeutic interventions and monitoring plans on their own experience and extrapolations from other addictive disorders comorbid with BD. Nevertheless, validated tools to quantify the severity of GD (five scales identified during this search) and psychotherapeutic interventions (mainly based on cognitive behavioral approaches) for GD have been identified and could be explored in further studies with BD patients. Based on the retrieved data, the use of structured psychometric instruments for measuring the severity of MDD and GD symptoms is needed, and we argue, based on data from studies that confirmed high rates of BD and addictive disorders, that the use of screening structured instruments for such addictive disorders, GD included, should be applied in patients with BD.

**Conclusions:**

Research on BD-GD comorbidity is still in its early phases, but instruments that could be investigated for screening and therapeutic purposes have been identified.

**Disclosure of Interest:**

None Declared

